# Decoding the aroma of *Rosa canina* L.: Chemical composition and gene expression

**DOI:** 10.1371/journal.pone.0316324

**Published:** 2025-01-24

**Authors:** Parisa Jariani, Ali-Akbar Shahnejat-Bushehri, Roohangiz Naderi, Meisam Zargar, Mohammad Reza Naghavi

**Affiliations:** 1 Division of Biotechnology, Department of Agronomy and Plant Breeding, College of Agricultural and Natural Resources, University of Tehran, Karaj, Iran; 2 Department of Horticulture Science, College of Agriculture and Natural Resources, University of Tehran, Karaj, Iran; 3 Department of Agrobiotechnology, Institute of Agriculture, RUDN University, Moscow, Russia; University of Kashan, ISLAMIC REPUBLIC OF IRAN

## Abstract

**Objective:**

The aromatic profile of *Rosa canina* L. petals hold immense potential for the fragrance and pharmaceutical industries. This study aims to investigate the chemical composition and gene expression patterns across different floral development stages to uncover the biosynthetic pathways of floral scent.

**Methods:**

Essential oils (EOs) were extracted from petals at five developmental stages (S1-S5) and analyzed using Gas Chromatography-Mass Spectrometry (GC-MS), identifying 20 distinct compounds. RNA isolation and quantitative real-time PCR (qRT-PCR) analysis were performed to assess gene expression.

**Results:**

Stage S3, notable for its enhanced aromatic profile, was dominated by terpenoid compounds such as β-Citronellol (1.18%), Caryophyllene (8.59%), β-Selinene (1.50%), and Caryophyllene oxide (0.50%), indicating significant upregulation of terpenoid biosynthesis genes. qRT-PCR analysis revealed that *CCD1* had the highest expression in S4 (9.51-fold), while *DXR* and *DXS* peaked at S3 with fold changes of 29058.38 and 73.35, respectively. Other genes like *AAT1*, *LIS*, and *GPS* also showed peak expressions at S3 with fold changes of 1.33, 10.70, and 1.18, respectively. *PAR* exhibited the highest expression in S1, while *GGPPS* peaked in S4 (2.01-fold). Clustering analysis indicated distinct groupings of developmental stages and gene expression patterns, with strong correlations between specific genes and compounds, such as *CCD1* with *GGPPS* (0.78) and β-Citronellol with Caryophyllene (0.92). Principal Component Analysis (PCA) highlighted significant contributions of *AAT1*, *GPS*, and nonadecane compounds to the overall variance.

**Conclusion:**

These findings provide a comprehensive understanding of the chemical and genetic factors shaping the aromatic profile of *R*. *canina*, with promising applications for both the fragrance and pharmaceutical sectors. The study’s innovation lies in the detailed correlation between EO composition and gene expression, presenting new insights into the biosynthetic pathways of floral scent.

## Introduction

*Rosa canina* L., commonly known as the dog rose, has captivated attention not only for its delicate petals and subtle fragrance but also for its rich history in traditional medicine and modern applications [1, 2]. This wild rose species offers a unique opportunity for scientific exploration, particularly in understanding its biochemical and genetic complexity [3].

Despite the recognized medicinal and aromatic properties of *R*. *canina*, comprehensive research on the biochemical composition and gene expression involved in its terpenoid biosynthesis remains limited. Previous studies have primarily focused on cultivated rose varieties, with less emphasis on wild species like *R*. *canina* [4]. This study aims to fill this gap by exploring the phenotypic and biochemical evolution of *R*. *canina* flowers across five developmental stages (S1 to S5), specifically examining changes in essential oil composition and gene expression.

In the field of EOs, *R*. *canina* exemplifies the complexity and diversity of plant-derived compounds [5]. Each developmental stage of the flower, from bud to full bloom, holds a unique aromatic signature, shaped by various biochemical pathways [6]. Understanding these volatile compounds, from their biosynthesis to their extraction and analysis, reveals intricate molecular interactions defining the essence of *R*. *canina*.

Essential oils from plants have been extensively studied for their biological activities, including antimicrobial, antioxidant, and anti-inflammatory properties [7–9]. The extraction and analysis of EOs from *R*. *canina* at different developmental stages can provide valuable insights into the dynamic changes in their chemical composition. Gas Chromatography-Mass Spectrometry (GC/MS) is a powerful technique used to identify and quantify volatile compounds in essential oils [10–12].

The scent of rose flowers, including *R*. *canina*, is primarily attributed to a mixture of volatile organic compounds, with key contributors such as geraniol, β-citronellol, and rose oxide [13, 14]. Geraniol, a major component, is responsible for the sweet and floral aroma of roses [15, 16]. The biosynthesis of these compounds involves several enzymes, including farnesyl diphosphate (FPP) synthase and NUDX1 hydrolase, crucial for producing geraniol and other scent-related compounds [17].

The terpenoid biosynthesis pathway is a significant metabolic route in plants, responsible for producing a wide range of volatile organic compounds that contribute to the aroma and therapeutic properties of EOs [18–21]. The interplay between flower color and scent in roses is an intriguing area of study, as these traits often work synergistically to attract pollinators and enhance reproductive success. The vibrant colors of rose petals, resulting from pigment accumulation like anthocyanins, contribute to the flowers’ visual appeal and signal to pollinators. Simultaneously, the release of volatile organic compounds serves as an olfactory cue, further enticing pollinators. This synergistic effect of color and scent enhances the overall attractiveness of flowers, increasing the likelihood of successful pollination and seed production [22].

This study aims to explore the phenotypic and biochemical evolution of *R*. *canina* flowers across five developmental stages (S1 to S5), focusing on essential oil composition and gene expression changes. These findings are expected to enhance our understanding of plant metabolism and its applications in aromatherapy, perfumery, and pharmaceuticals.

## Material and methods plant materials

This study utilized white flowers of *Rosa canina* L. from five distinct floral development stages (S1-S5), endemic to the mountainous regions of Taleghan, Alborz Province, Iran. The flowers were collected from a wild population located at 36°10′58″ N and 50°52′50.2″ E. A voucher specimen (number 006962) was deposited in the Herbarium Instituti Agronomici KeredJensis (HIAK) by a botanist from the horticulture department of the University of Tehran (Dr. Vahideh Nazeri Jounghani). Identification was performed based on morphological characteristics of leaves, fruits, and flowers, following the methods described in reference [5].

Due to the constraints of working with wild plants, one shrub was selected and three technical replicates were prepared for each stage. This approach was taken to maintain consistency and reliability within the selected specimen, recognizing the limitations in obtaining biological replicates.

The flowers were harvested in the morning at 7:00 a.m., a time previously determined to be optimal for volatile oil content [23–25]. Samples were obtained from the same shrub, and three technical replicates were prepared for molecular analysis, resulting in a total of 30 flowers being collected to ensure a representative sample across all developmental stages.

**Fig 1:**
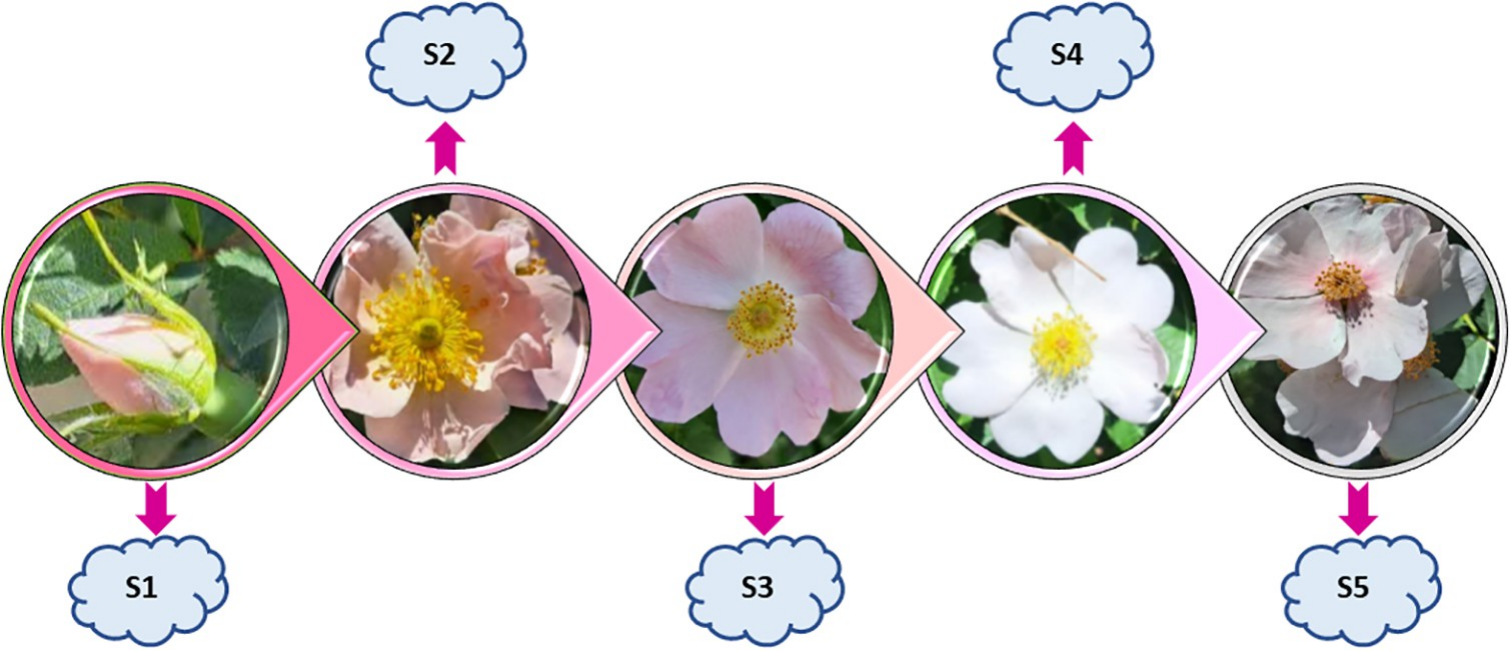
Floral development stages of *R*. *canina* L. This figure depicts the morphological changes in the flower from bud initiation to senescence. The stages are labeled as follows: S1, budding stage; S2, early opening stage; S3, newly opened stage; S4, fully opened stage; S5, senescence stage.

The flowers were collected at five different critical stages of floral development for scent emission:

1. Budding Stage (S1): Sepals were closed but petal colors were visible.
2. Early Opening Stage (S2): Pollen was light yellow; petals were fresh and pale pink but not fully open, showing semi-closed petals.
3. Newly Opened Stage (S3): Petals were paler pink; pollen was fresh but slightly darker than in S2.
4. Fully Opened Stage (S4): Petals were fully open and had lightened to white; pollen was very dark.
5. Senescence Stage (S5): Petals were completely white and curled both inside and outside; pollen was minimal and sometimes black or dark brown, and petals were weak and easily detached (Fig 1) [26–28].

To prevent RNA degradation, fresh samples were immediately frozen in liquid nitrogen and stored at −80°C until further analysis. The choice of treatment stages and harvesting times was based on established protocols [3, 5, 25] to ensure optimal volatile oil content and accurate molecular analysis.

### Extraction of Essential Oils (EOs)

To extract EOs from *Rosa* petals, fresh petals were meticulously separated from other floral parts [29]. Approximately 200 g of fresh petals (S1–S5) were weighed for the extraction process. The EOs were extracted using the hydrodistillation method, which involves heating water and plant material in a closed system and condensing the vapor containing the volatile compounds [30–33]. This method was selected due to its simplicity, cost-effectiveness, and widespread use for extracting EOs from various plants [34].

The extraction was conducted using a 2 L Clevenger apparatus over a period of 4 hours. The extracted EOs were then stored in dark glass vials at a low temperature of −4°C to prevent evaporation and loss of the oils until gas chromatography-mass spectrometry (GC-MS) analysis [35].

### GC/MS analysis

Gas chromatography (GC) was performed using an Agilent 7890 instrument, coupled with a 5977-mass spectrometer (MS) detector, to ensure precise separation and identification of the sample components [36]. An HP-5 MS capillary column (30 m length x 0.25 mm internal diameter x 0.25 μm film thickness) was employed for the GC-MS analysis, providing high-resolution separation of the analytes [37]. The analysis was conducted using splitless injection to maximize sample introduction efficiency. The initial column temperature was set at 60°C with no hold time. The temperature was programmed to increase at a rate of 5°C per minute until reaching a final temperature of 250°C, which was maintained for 10 minutes to ensure complete elution of all analytes. A 1 μL sample was injected into the port, where it was immediately vaporized and carried through the column by ultra-pure helium at a flow rate of 1 mL/min, ensuring consistent and reproducible results. The injector temperature was maintained at 250°C to facilitate efficient sample vaporization. The MS spectrum was acquired at 70 eV, providing detailed mass spectral data for compound identification.

### Identification of compounds

After chromatographic separation, the components were identified and analyzed using mass spectrometry. The identification process involved comparing the spectra of unknown compounds with those in the Wiley and NIST MS 2.0 structural libraries [38, 39]. This comparison enabled the determination of compound names, molecular weights, and structures, ensuring accurate and reliable identification of the sample constituents. The identified compounds and their average area percentages for three replicates (%) are presented in Table 1.

**Table 1:** Percentage composition of the essential oils isolated from white *R*. *canina* petals at different floral development stages (S1 to S5). Data represent the mean of three replicates.

Compound number	Compound name	RT (min)	Percentage Peak Area%				
			S1	S2	S3	S4	S5
1	Decane	6.156	7.16	1.112	0.963	0.511	2.112
2	Undecane	8.50	8.99	1.035	2.89	0.427	1.001
3	Dodecane	11.08	2.94	7.051	0.460	0.237	1.030
4	beta. -Citronellol	11.86	.	1.143	1.182	.	2.797
5	Caryophyllene	16.87	1.330	3.755	8.586	4.276	24.102
6	Naphthalene	18.50	.	1.522	.	1.417	0.544
7	beta.-Selinene	18.53	.	2.667	1.498	.	0.764
8	Caryophyllene oxide	20.832	1.33	.	0.503	.	1.968
9	9-Nonadecene	26.95	5.865	1.358	3.59	1.312	0.908
10	Z-5-Nonadecene	26.99	5.87	2.215	3.591	0.945	0.908
11	Nonadecane	27.50	2.78	2.534	3.791	1.599	1.536
12	Hexadecenoic acid	27.94	2.86	.	.	5.435	0.642
13	Eicosane	29.39	8.96	0.5	0.941	0.584	1.030
14	Heneicosane	31.35	29.07	7.807	10.170	11.455	7.163
15	Docosane	33.03			1.026	1.497	1.057
16	Tricosane	34.84	11.14	6.618	6.378	19.107	14.430
17	Pentacosane	37.98	.	2.928	3.298	.	.
18	Hexacosane	39.61	.	.	.	0.617	0.399
19	Heptacosane	41.73	29.069	1.574	0.54	9.038	6.897
20	Nonacosane	47.41	.	.	10.317	1.643	1.084

### RNA isolation and quantitative real-time PCR (qRT-PCR)

**Fig 2:**
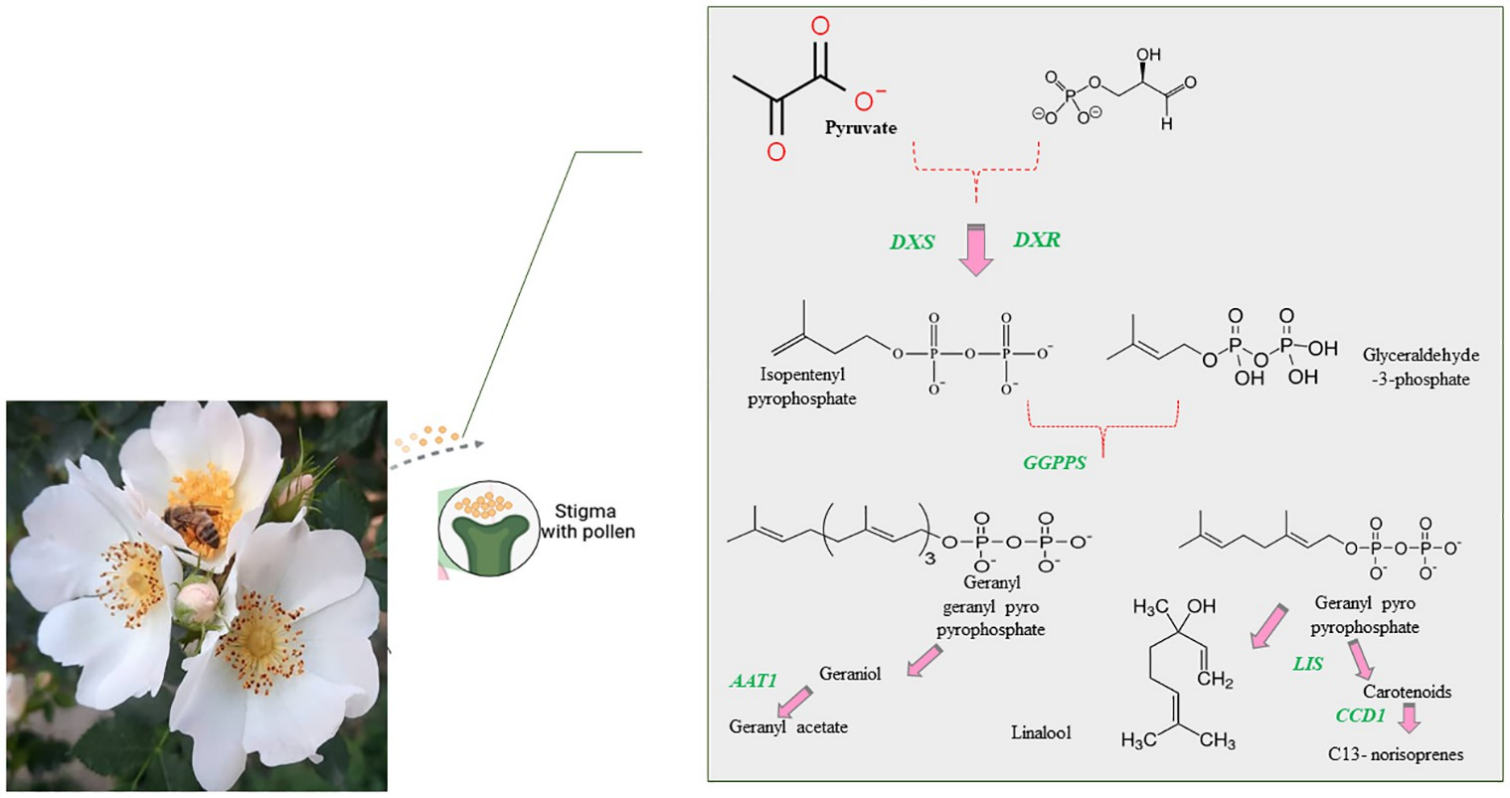
Detailed schematic of the MEP (2-C-methyl-D-erythritol 4-phosphate) pathway involved in terpenoid biosynthesis in roses. This pathway includes key intermediates and enzymes such as G3P (glyceraldehyde-3-phosphate), *DXS* (1-deoxy-D-xylulose-5-phosphate synthase), *DXR* (1-deoxy-D-xylulose 5-phosphate reductoisomerase), *LIS* (linalool synthase), *AAT1* (aromatic amino acid aminotransferase), and *CCD1* (carotenoid cleavage dioxygenase 1). The diagram illustrates the biochemical processes leading to the formation of various terpenoids, which are crucial for the aromatic properties and other biological functions in roses.

To elucidate the biosynthetic pathways of key genes involved in flower scent, particularly terpenoid biosynthesis (as depicted in Fig 2), gene expression profiling was conducted [40, 41]. Approximately 200 mg of petal tissues were cryogenically pulverized using liquid nitrogen. Total RNA was extracted using an optimized cetyltrimethylammonium bromide (CTAB) protocol [42]. Residual genomic DNA was removed with *DNase I* treatment using the RNase-Free DNase Set (Fermentas, Waltham, MA). The RNA’s purity and quantity were verified by agarose gel electrophoresis and spectrophotometric analysis with a Nanodrop ND-1000 instrument [43].

For complementary DNA (cDNA) synthesis, 1 μg of total RNA was reverse transcribed using the A101161 Reverse Transcription Kit (Parstous, Iran), following the manufacturer’s instructions. Primers for genes such as *PAR*, *GGPPS*, *GPS*, *DXR*, *DXS*, *LIS*, *AAT1*, and *CCD1* were designed using Primer3 software, Gene Runner, and the online Oligo Analyzer tool. Primer efficiency was confirmed using the Oligo Analyzer tool, Gene Runner software, and NCBI/Primer-BLAST.

The beta-actin gene served as an internal control to normalize target gene expression levels. qRT-PCR assays were conducted on QIAGEN’s real-time PCR system, adhering to the manufacturer’s guidelines. Each 15 μL reaction mixture contained 7.5 μL of SYBR Green Master Mix 2X (ROX), 4.7 μL of RNase-free water, 2 μL of the cDNA template, and 0.4 μL each of forward and reverse primers. Thermal cycling conditions included an initial denaturation at 95°C for 15 minutes, followed by 40 cycles of 15-second denaturation at 95°C, 20-second annealing at 56–59°C (primer-specific optimal temperatures determined by gradient PCR), and 20-second extension at 72°C.

### Statistical analysis

Relative mRNA expression levels were quantified using the 2^-ΔΔCT^ method [44–46]. Statistical analysis of gene expression data was performed using R software (version 4.3) and RStudio, based on a completely randomized design (CRD). Duncan’s multiple range test determined the significance of mean differences at 5% (P≤0.05) and 1% (P≤0.01) probability levels.

Gene expression patterns were visualized using ORIGIN PRO software. Clustering of gene expression data was performed using the R package for cluster analysis. The resulting dendrogram was depicted as a heatmap, employing Ward’s minimum variance method to determine the distance metric. The color gradation within the heatmap represented the correlation between biosynthetic pathways responsible for flower scent and floral development stages.

### Correlation analysis between GC-MS and qRT-PCR results and Principal Component Analysis (PCA)

PCA analysis was employed to reduce the dimensionality of the dataset and to identify the most influential variables [47–49]. The analysis was conducted using the R Studio. To explore the correlation between gene expression and compound abundance, a comprehensive analysis was performed using R Studio software. Correlation coefficients were calculated to determine significant associations between the expression levels of specific genes and the abundance of corresponding compounds.

## Results

### GC-MS profiling of *R*. *canina* petals EOs

This study conducted an in-depth GC-MS analysis of EOs extracted from the white petals of *R*. *canina* at various stages of floral development, using hydrodistillation performed in triplicate. A total of 20 distinct compounds were identified across the developmental stages. Stage S3 was particularly notable for its enhanced aromatic profile, dominated by terpenoid compounds such as β-Citronellol, Caryophyllene, β-Selinene, and Caryophyllene oxide. This suggests an upregulation of gene expression in the terpenoid biosynthetic pathway during this phase.

The identified compounds were categorized based on carbon numbering and classified into four main classes: terpenoids, aliphatic hydrocarbons, aldehydes/ketones, and alcohols, showcasing a diverse chemical profile suitable for various applications. Retention indices (RI) and linear retention indices (LRI) were employed for compound identification, ensuring accurate and reliable results. The average concentrations of the most abundant compounds across five floral stages are presented in Table 1.

### RNA isolation and quantitative real-time PCR (qRT-PCR)

**Fig 3:**
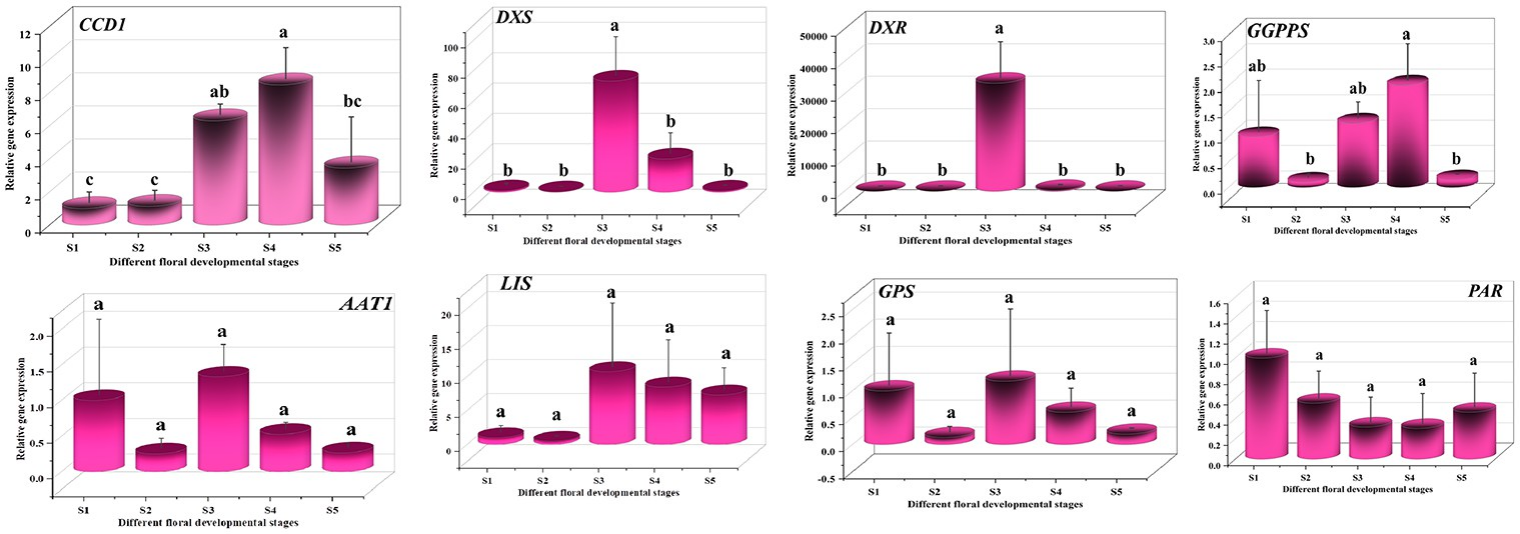
Differential expression of key genes in terpene biosynthesis pathways during *R*. *canina* L. flower development.

This study utilized qRT-PCR to measure and compare the relative expression levels of eight key genes involved in the scent production of white *R*. *canina* flowers. These genes, integral to the biosynthetic pathways responsible for floral scent, were examined across five distinct stages of floral development (S1 through S5). The results indicated that *CCD1* had the highest expression in S4 (9.51-fold), while *DXR*, *DXS*, *AAT1*, *LIS*, and *GPS* showed their highest expressions in S3, with fold changes of 29058.38, 73.35, 1.33, 10.70, and 1.18, respectively. *PAR* exhibited the highest expression in S1, and *GGPPS* had the highest expression in S4 (2.01-fold). Significant differences in gene expression were observed for *CCD1* in S4, followed by S3, for *DXS* and *DXR* in S3, and for *GGPPS* in S4, followed by S3 and S1 (Fig 3).

### Clustering analysis

The clustering analysis, visualized through a heatmap, revealed distinct groupings among the stages and genes involved in the scent production of white *R*. *canina* flowers. The stages exhibited clear clustering patterns, with S3 and S4 forming one cluster, while S2 and S5 formed another cluster, which subsequently joined with S1. Ultimately, the group consisting of S1, S5, and S2 merged with the group of S3 and S4. This hierarchical clustering suggests that the stages share common gene expression profiles, which may be indicative of similar metabolic activities or regulatory mechanisms during these stages.

Regarding the genes, the clustering analysis revealed that *DXS* and *DXR* clustered together, indicating a close functional relationship between these genes in the biosynthesis pathway. Similarly, *AAT1* and *GPS* formed another cluster, suggesting a potential co-regulation or interaction between these genes. These two clusters then merged, followed by the *PAR* gene joining this group, further highlighting the interconnectedness of these genes in the metabolic network. *LIS* and *CCD1* clustered together, with *GGPPS* joining this group, indicating a potential regulatory relationship or shared biosynthetic pathway among these genes. Finally, these clusters combined into a single group, suggesting a comprehensive network of interactions among the genes involved in scent production.

**Fig 4:**
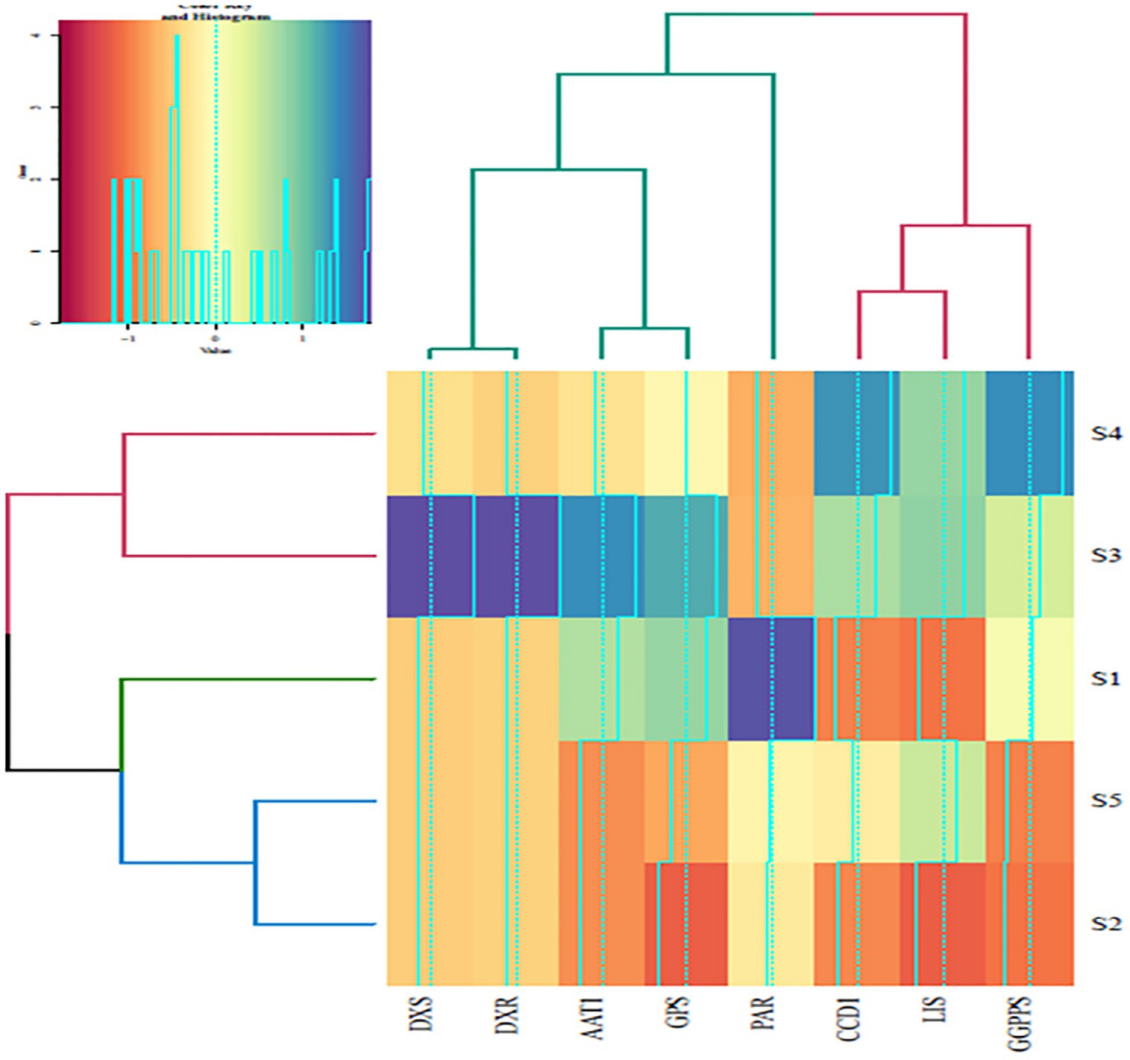
Heatmap and cluster analysis of gene expression in different floral developmental stages (S1 -S5) of *R*. *canina*.

The heatmap indicated high expression levels of *GGPPS* and *CCD1* in S4, marked by high blue intensity. This suggests that these genes play a significant role in the scent production during this stage. Similarly, *DXR*, *DXS*, *AAT1*, and *GPS* exhibited higher expression levels in S3, also indicated by high blue intensity, suggesting their importance in the metabolic activities during this stage. The *PAR* gene showed high expression in S1, indicating its potential role in the early stages of scent production Overall, the clustering analysis provided valuable insights into the gene expression patterns and their relationships during the different stages of scent production in white *R*. *canina* flowers. These findings can inform further research on the regulatory mechanisms and metabolic pathways involved in scent production, contributing to a deeper understanding of the underlying biological processes (Fig 4).

This figure depicts the relative gene expression levels involved in the biosynthesis of flower scent across five distinct stages of floral development in *R*. *canina* L. The morphological changes from bud initiation (S1) to senescence (S5) are captured, with stages designated as S1 (budding stage), S2 (early opening stage), S3 (newly opened stage), S4 (fully opened stage), and S5 (senescence stage). Gene expression fold changes were normalized to the *beta-actin* gene, with the S1 serving as the calibrator for white petal comparison. Error bars represent standard errors (mean ± SE, n = 3). Statistically significant differences between different floral development stages S1-S5 are denoted by distinct letters, with p-values less than 0.05, as determined by one-way ANOVA followed by Duncan’s multiple range test.

### Correlation analysis between GC-MS and qRT-PCR results

The correlation analysis conducted using R Studio software revealed several significant associations between gene expression and compound abundance, providing valuable insights into the underlying biological processes. Notably, *CCD1* exhibited a high correlation with *GGPPS* (0.78) and *LIS* (0.89), suggesting a potential regulatory relationship between these genes and their associated metabolic pathways. Beta-Citronellol showed a strong correlation with Caryophyllene (0.92), indicating a possible interaction or co-regulation in the biosynthesis of these compounds.

The *DXS* gene was highly correlated with Nonadecane (0.71), while *DXR* and *DXS* demonstrated a very high correlation (0.98), highlighting the close functional relationship between these genes in the isoprenoid biosynthesis pathway. Eicosane and Undecane were also strongly correlated (0.97), suggesting a shared biosynthetic origin or regulatory mechanism.

Further analysis indicated that Heneicosane had high correlations with Undecane (0.95) and Eicosane (0.98), while Heptacosane showed significant correlations with Heneicosane (0.95), Undecane (0.86), and Eicosane (0.95). These findings suggest that these compounds may be co-regulated or share common biosynthetic pathways.

Decane exhibited high correlations with Heptacosane (0.93), Heneicosane (0.92), Undecane (0.94), and Eicosane (0.98), indicating a potential network of interactions among these compounds. The *PAR* gene was strongly correlated with Undecane (0.92), Eicosane (0.97), Heneicosane (0.90), Heptacosane (0.92), and Decane (0.99), suggesting its involvement in the regulation of these compounds.

Additionally, the *AAT1* gene showed high correlations with Nonadecane (0.82), *DXS* (0.77), and *DXR* (0.78), indicating a potential role in the regulation of these genes and their associated metabolic pathways. The *GPS* gene was highly correlated with *AAT1* (0.98), further supporting the regulatory relationship between these genes.

**Fig 5:**
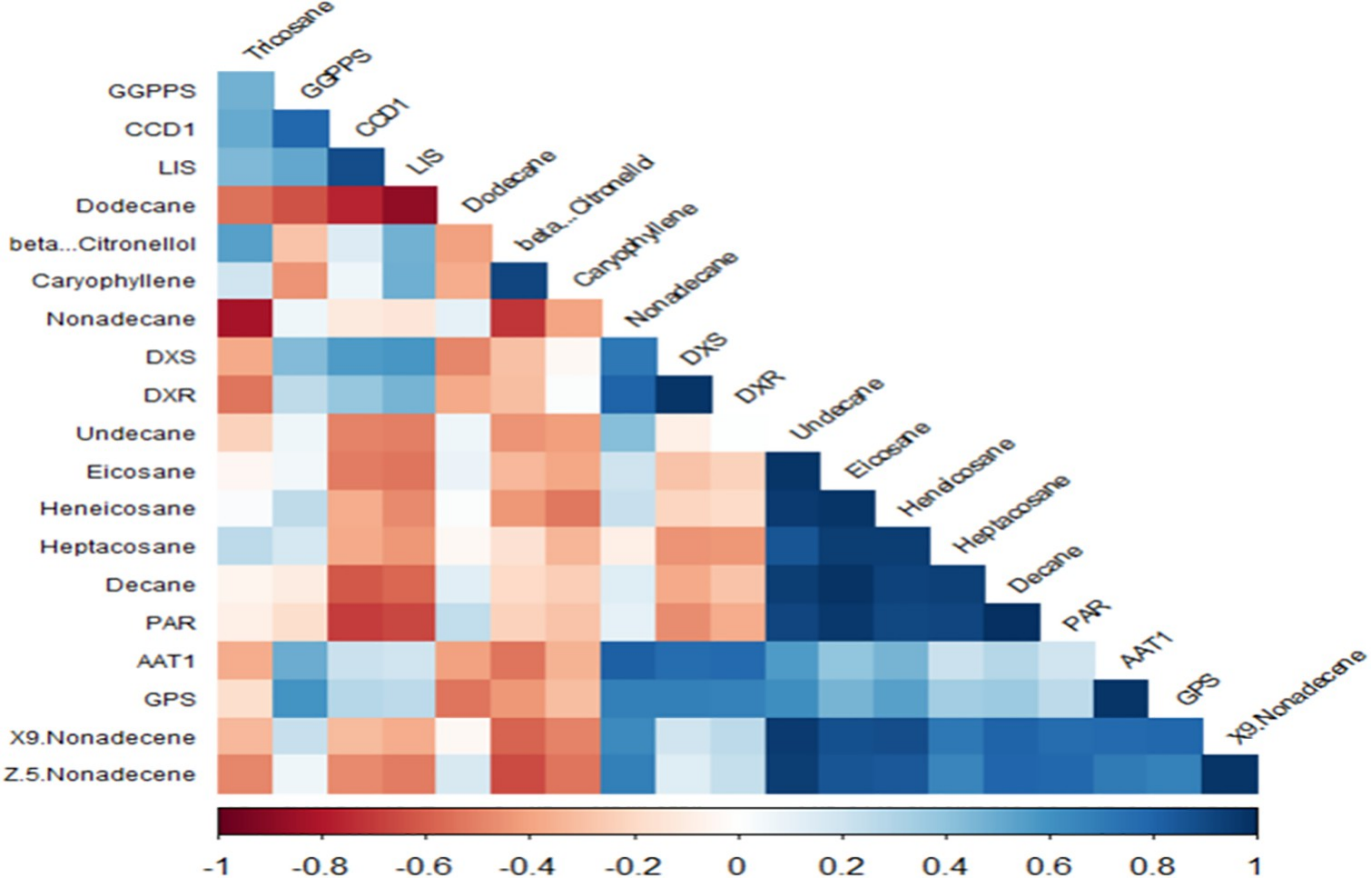
Correlation analysis between GC-MS and qRT-PCR results.

The X9. Nonadecane compound exhibited strong correlations with Undecane (0.96), Eicosane (0.87), Heneicosane (0.89), Heptacosane (0.72), Decane (0.81), *PAR* gene (0.77), *AAT1* (0.77), and *GPS* (0.79), suggesting a complex network of interactions among these compounds and genes. Lastly, Z.5.Nonadecane showed high correlations with Undecane (0.95), Eicosane (0.85), Heneicosane (0.85), Decane (0.80), *PAR* (0.78), *AAT1* (0.70), and 9. Nonadecane (0.98), indicating its involvement in the regulation of these compounds and genes (Fig 5).

### 3.4. Principal Component Analysis (PCA) analysis

The PCA conducted on the dataset provided significant insights into the underlying structure of the data [50–52]. The first principal component (Dim 1) accounted for 44.4% of the total variance, highlighting its critical role in explaining the variability within the dataset. This dimension was predominantly influenced by variables such as *AAT1*, *GPS*, X9 Nonadecane, and Z.5 Nonadecane, suggesting these variables are highly correlated and play a crucial role in this dimension. The high percentage of variance explained by Dim 1 indicates that these variables capture a substantial amount of the dataset’s information.

**Fig 6:**
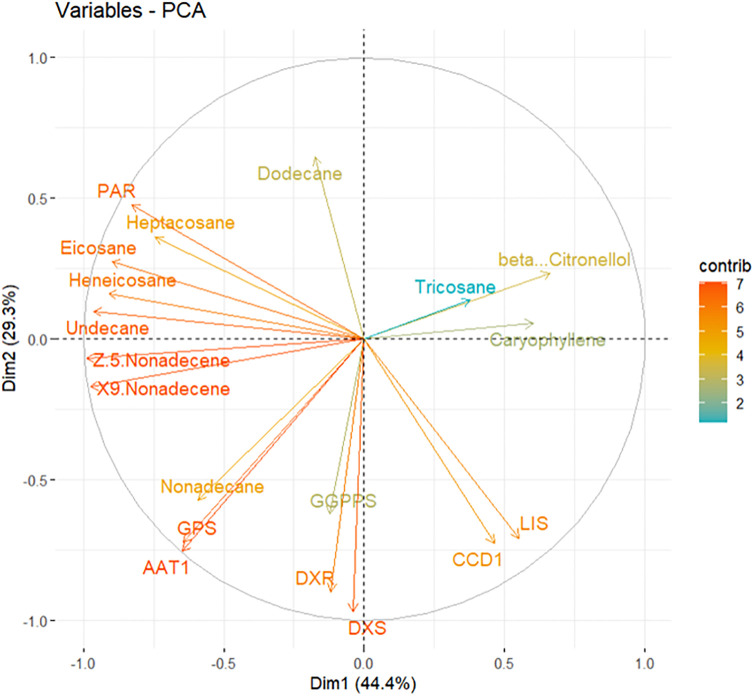
Principal Component Analysis (PCA) of chemical compounds and gene expression patterns.

The second principal component (Dim 2) explained 29.3% of the variance, further enhancing our understanding of the dataset’s structure. This dimension was characterized by strong positive correlations with Undecane, Henicosane, Eicosane, and the *PAR* gene, indicating these variables are key factors in Dim 2 and provide additional insights into the dataset’s variability (Fig 6).

## Discussion

Floral scent plays a crucial role in the reproductive success of flowering plants by attracting pollinators [53, 54]. The chemical composition and biosynthesis of floral volatiles are complex processes regulated by various genes [55, 56]. This study explores the floral scent production in *R*. *canina*, highlighting the key compounds and genetic mechanisms involved.

The relative expression levels of eight key genes involved in the scent production of white *R*. *canina* flowers were measured across five distinct stages of floral development (S1-S5). The results indicated significant upregulation of specific genes at different developmental stages, which aligns with their roles in the biosynthetic pathways responsible for floral scent. *CCD1* exhibited the highest expression in S4, while *DXR*, *DXS*, *AAT1*, *LIS*, and *GPS* showed their highest expressions in S3 with substantial fold changes, indicating a coordinated regulation during this stage. *PAR* had the highest expression in S1, and *GGPPS* peaked in S4, suggesting stage-specific regulatory mechanisms.

The high expression levels of genes such as *CCD1*, *DXR*, and *DXS* are crucial for the biosynthesis of terpenoids and other volatile compounds [57, 58], which are essential for the characteristic fragrance of *R*. *canina*. These findings are consistent with recent studies on other species, such as *R*. *chinensis* and *Petunia hybrida*, where similar patterns of gene expression have been observed [59–61].

The petals of *R*. *canina* are rich in a variety of volatile compounds, including terpenoids, aliphatic hydrocarbons, aldehydes/ketones, and alcohols [5]. Notably, β-Citronellol and Caryophyllene are major constituents. β-Citronellol is renowned for its pleasant floral scent and is a common component of rose oils, contributing significantly to their characteristic fragrance [62, 63]. Caryophyllene, a sesquiterpene, is known for its anti-inflammatory and analgesic properties, suggesting potential therapeutic applications for *R*. *canina* EOs [64, 65].

The biosynthesis of these volatile compounds is tightly regulated by gene expression. *CCD1* is involved in the carotenoid cleavage pathway, contributing to the production of volatile compounds. *DXR* and *DXS* are crucial in the MEP pathway, essential for terpenoid biosynthesis [66, 67]. Additionally, *LIS* was found to be a key gene in the biosynthesis of linalool, a major floral scent compound [68, 69]. The hierarchical clustering of gene expression during different stages of flower development suggests a coordinated regulation.

Correlation analysis revealed significant associations between gene expression and compound abundance. *CCD1* exhibited high correlations with *GGPPS* (0.78) and *LIS* (0.89), indicating potential regulatory relationships. β-Citronellol showed a strong correlation with Caryophyllene (0.92), suggesting co-regulation in their biosynthesis. *DXS* and *DXR* demonstrated a high correlation (0.98), highlighting their close functional relationship in the isoprenoid biosynthesis pathway. Further analysis indicated strong correlations among Eicosane, Undecane, Heneicosane, and Heptacosane, suggesting shared biosynthetic origins or regulatory mechanisms.

PCA provided significant insights into the dataset’s structure. The first principal component (Dim 1) accounted for 44.4% of the total variance, predominantly influenced by *AAT1*, *GPS*, X9 Nonadecane, and Z.5 Nonadecane. The second principal component (Dim 2) explained 29.3% of the variance, characterized by strong positive correlations with Undecane, Heneicosane, Eicosane, and the *PAR* gene. These analyses underscore the importance of specific genes and compounds in shaping the aromatic profile of *R*. *canina*.

Comparative studies reveal both conserved and unique aspects of scent production in *R*. *canina*. The expression of *PAR* in the early stages of scent production in *R*. *canina* contrasts with its role in *Arabidopsis thaliana*, highlighting species-specific regulatory mechanisms. These comparisons underscore the importance of studying different species to uncover diverse regulatory mechanisms involved in floral scent biosynthesis.

Understanding the genetic and chemical basis of floral scent can inform further research and potential applications in various industries, including cosmetics and therapeutics. Future research should focus on the functional characterization of the identified compounds and their biosynthetic pathways to further elucidate the molecular mechanisms underlying floral scent formation.

## Conclusion

This study unveils the intricate relationship between the chemical composition and gene expression patterns of *R*. *canina* petals, offering groundbreaking insights into the biosynthetic pathways of floral scent. By correlating the stages of floral development with the aromatic profile, we have identified key terpenoid compounds and their associated genes, shedding light on the dynamic nature of scent biosynthesis. The significant upregulation of specific genes during peak aromatic stages underscores the potential for targeted genetic manipulation to enhance desirable traits.

The novelty of this research lies in its holistic approach, integrating chemical analysis with gene expression profiling to provide a comprehensive understanding of the factors influencing floral scent. These findings pave the way for innovative applications in the fragrance and pharmaceutical industries, where the manipulation of scent profiles can lead to the development of new, high-value products. Future research should focus on exploring the functional roles of these compounds and genes in other *Rosa* species, as well as investigating the environmental factors that may influence gene expression and compound biosynthesis. This study sets the stage for the development of new *Rosa* varieties with enhanced aromatic properties, offering exciting possibilities for both commercial and scientific advancements. Future research should explore the functional roles of these compounds and genes in other *Rosa* species, potentially leading to the development of new varieties with enhanced aromatic properties.

## Supporting information

S1 Data (XLSX)
